# Role of CpG deserts in the epigenetic transgenerational inheritance of differential DNA methylation regions

**DOI:** 10.1186/1471-2164-15-692

**Published:** 2014-08-20

**Authors:** Michael K Skinner, Carlos Guerrero-Bosagna

**Affiliations:** Center for Reproductive Biology, School of Biological Sciences, Washington State University, Pullman, WA 99164-4236 USA

**Keywords:** CpG density, DNA methylation, Transgenerational, Genomic feature, CpG desert

## Abstract

**Background:**

Previously a variety of environmental toxicants were found to promote the epigenetic transgenerational inheritance of disease through differential DNA methylation regions (DMRs), termed epimutations, present in sperm. The transgenerational epimutations in sperm and somatic cells identified in a number of previous studies were further investigated.

**Results:**

The epimutations from six different environmental exposures were found to be predominantly exposure specific with negligible overlap. The current report describes a major genomic feature of all the unique epimutations identified (535) as a very low (<10 CpG/100 bp) CpG density in sperm and somatic cells associated with transgenerational disease. The genomic locations of these epimutations were found to contain DMRs with small clusters of CpG within a general region of very low density CpG. The potential role of these epimutations on gene expression is suggested to be important.

**Conclusions:**

Observations suggest a potential regulatory role for lower density CpG regions termed “CpG deserts”. The potential evolutionary origins of these regions is also discussed.

## Background

The identification of DNA methylation at cytosines adjacent to guanine (CpG) sites was the first epigenetic mechanism and mark established [1, 2]. The initial restriction enzyme based DNA methylation analysis was biased to high density CpG regions that were subsequently referred to as CpG islands [3]. Functional studies of the role of these CpG islands has lead to the dogma that they are the regulatory regions for DNA methylation. More recent marks such as 5 hydroxymethyl-cytosine [4] have expanded our understanding of the DNA methylation. The technology to investigate DNA methylation has advanced such that genome-wide DNA methylation profiling and mapping is now feasible [5]. Although CpG islands have been thought to be the primary regions to regulate gene expression [3], more recent data suggests that the lower density CpG shores of islands may be important [6]. Genome-wide epigenetic studies have also suggested that low density CpG regions of the genome appear more regulatory than previously considered [7]. CpG-rich and CpG-poor promoters appear regulated differentially, not only by DNA methylation but also by the polycomb system [8]. Recent literature suggests that lower density regions may be more important for distal regulation of gene expression [9] through regulatory elements such as enhancers [10]. High density CpG regions such as CpG islands appear to regulate genome activity in house keeping and tissue specific genes [8]. Although the low and high CpG density regions are critical for the regulation of genome activity, both appear to have distinct functions.

Our laboratory has demonstrated that a variety of environmental toxicants can promote the epigenetic transgenerational inheritance of disease and phenotypic variation [11, 12]. The molecular mechanism involved is that exposure of a gestating female during the period of fetal gonadal sex determination promotes the altered epigenetic programming of the germline which appear to become permanently programmed and transmit this altered epigenome to subsequent generations [12]. The epigenetic transgenerational inheritance phenomena is defined as “germline mediated transmission of epigenetic information between generations in the absence of direct exposures or genetic manipulations” [12]. All the environmental toxicants studied have been shown to promote altered differential DNA methylation regions (DMR) in the sperm, termed epimutations [13]. Interestingly, the epimutation signatures observed in the sperm are exposure specific with negligible overlap between the specific epimutations [13]. Characterization of these DMR and epimutation signatures has identified a major genomic feature associated with these DMR is a very low (<10 CpG/100 bp) CpG density [13, 14]. The current report examined the epimutations previously identified in ten different studies to provide a novel perspective on the lower density CpG regions in the genome. We refer to these regions as “CpG deserts”.

## Results

**Figure 1:**
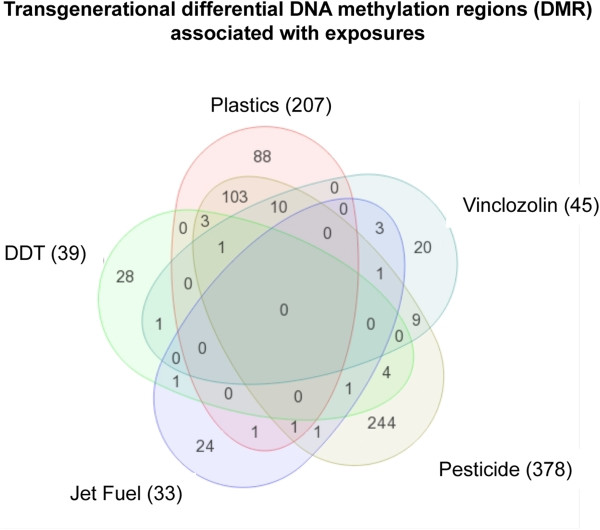
**Transgenerational F3 generation sperm epimutations specificity and overlap.** A Venn diagram of DMR from various F3 generation exposure lineages including: vinclozolin, plastics (BPA and phthalates), pesticides (permethrin and DEET), hydrocarbons (JP8) and DDT. The total number of DMR per exposure lineage in brackets is presented and unique and overlapping DMR identified. The total number of epimutations listed had overlapping DMR considered, so n = 535 unique epimutations (p < 10^-5^) were investigated in the current study. Modified from [13].

The previously identified DMR for the transgenerational sperm epimutations involved a number of different studies [13] and data sets including the F3 generation sperm epimutations from vinclozolin [14], plastics compounds (bisphenol A (BPA) and phthalates) [15], hydrocarbons (JP8 jet fuel) [16], pesticides (permethrin and DEET [N,N-diethyl-meta-toluamide]) [17], dioxin [18] and DDT (dichlorodiphenyltrichloroethane) [19] exposure lineages. The total number of unique transgenerational sperm epimutations examined is 535 DMR. Each individual DMR was identified with a methylated DNA immunoprecipitation (MeDIP) procedure followed by a genome wide promoter tiling array (MeDIP-Chip) protocol [13–19]. A statistical difference in DNA methylation between the F3 generation control lineage versus the F3 generation exposure lineage sperm used a p-value of p < 10^-5^ [13–19]. The average size of the DMR was 500-1,500 bp for the sperm DMR identified [13, 14]. The number of DMR (i.e. epimutations) associated with each specific exposure in the F3 generation sperm is shown in Figure 1. Interestingly, none of the DMR were found to overlap between all exposures [13, 19] and the majority were exposure specific, Figure 1.

**Figure 2:**
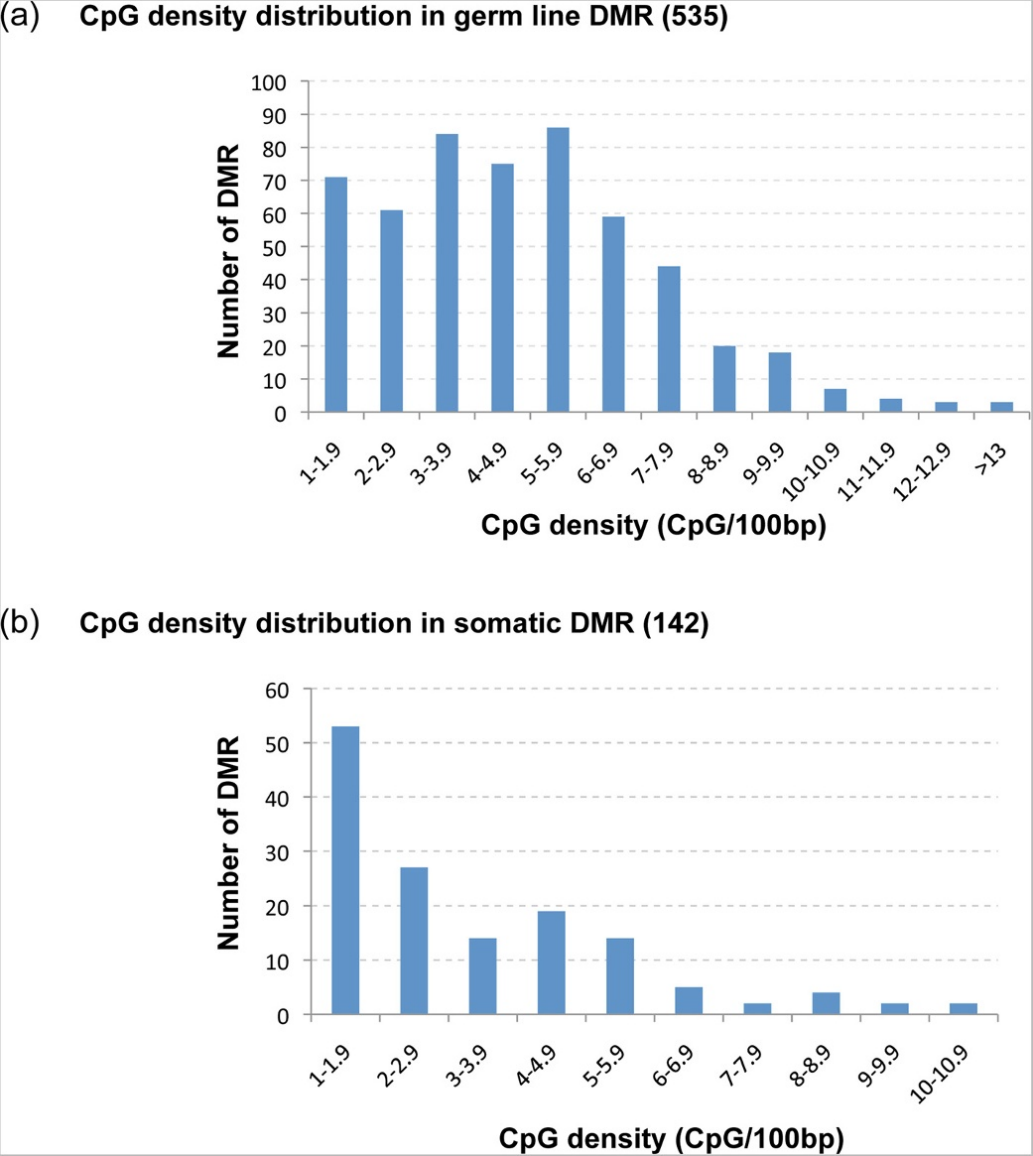
**Transgenerational differential DNA methylation regions (DMR) CpG density and association with CpG deserts. (a)** CpG density distribution in n = 535 distinct sperm DMR with the number of DMR correlated with variable CpG density (CpG/100 bp). **(b)** CpG density distribution in n = 142 distinct somatic cell (Sertoli and granulosa cells) DMR with the number of DMR correlated with variable CpG density (CpG/100 bp).

The CpG density of each of the DMR for all the treatments was identified using the number of CpG/100 bp to determine the density. Analysis of the CpG density for these sperm DMR is shown in Figure 2a and indicates a density <13 CpG/100 bp with the majority of DMR having 1 to 8 CpG/100 bp density. Greater than 97% of the DMR had a <10% CpG density. These CpG deserts of <15 CpG/100 bp, termed CpG deserts, are present in all the sperm DMR examined [13–19], Figure 2a. Therefore, the CpG density of the epimutations was very low.

In addition to the analysis of sperm epimutations, the somatic cell transgenerational DMR for testicular Sertoli cells associated with testis disease [20] and ovarian granulosa cells associated with ovarian disease [21] from F3 generation vinclozolin lineage animals were identified. The epimutations were also selected based on a statistically significant difference of p < 10^-5^ [20, 21]. The CpG density for these somatic cell DMR was also <10 CpG/100 bp, Figure 2b. These somatic cell transgenerational DMR had negligible overlap with sperm DMR and with each other, such that they were cell specific [20, 21]. Therefore, both the transgenerational sperm and somatic cell epimutations had DMR that were “CpG deserts”.

**Figure 3:**
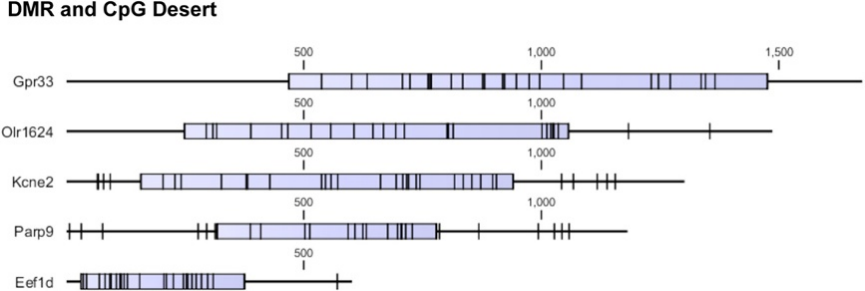
**Genomic mapping of selected gene F3 generation vinclozolin lineage sperm promoter DMR with blue box indicating the region with differential DNA methylation and specific CpG residues (black hatch marks) for variable base pair length regions.**

**Figure 4:**
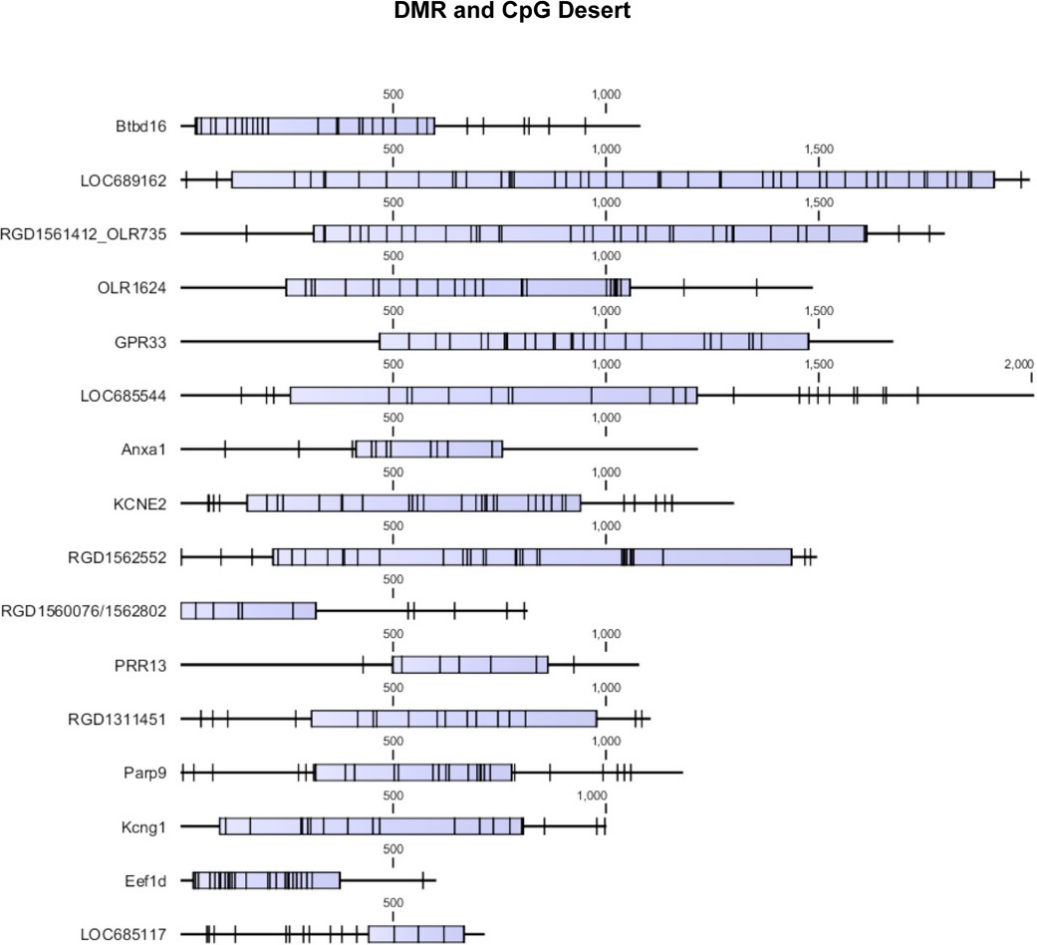
**Genomic mapping of selected F3 generation vinclozolin lineage sperm gene promoter DMR with blue box indicating the region with differential DNA methylation and specific CpG residues (black hatch marks) for variable length base pair regions.**

The detailed CpG density maps for selected sperm epimutations are shown in Figure 3. The F3 generation vinclozolin lineage sperm DMR are presented for the promoters of *Gpr33, Olr1624, Kcme2, Parp9* and *Eef1d*. The blue box represents the DMR region with statistically significant (p < 10^-5^) altered CpG methylation and the black hatch marks represent individual CpG sites within the DMR that are identified as 500 to 1500 bp in length, Figure 3. The low density CpG within these CpG deserts can be observed and the presence of small clusters of CpG within the DMR are indicated. No CpG islands were observed within these 500 to 1500 bp regions. Additional examples of transgenerational sperm DMR and CpG deserts are presented in Figure 4 for a variety of different gene promoters with varying size (500 to 2000 bp). The low density CpG and small CpG clusters containing a few CpG sites can be seen in all the sperm epimutations for these CpG deserts. In addition to this low density genomic feature (i.e. CpG desert), unique DNA sequence motifs have also recently been observed within these DMR [14].

## Discussion

Consideration of the genomic features of the transgenerational sperm and somatic cell epimutations identified the existence of CpG deserts containing small clusters of CpG within the DMR. These epimutations are potential regulators of genome activity and are involved in the epigenetic transgenerational inheritance phenomenon. Germline epimutations are critical in mediating the transmission of altered epigenetic information between generations [12]. All tissues and cells derived from this altered germline epigenome will have an altered epigenome and transcriptome [20–22]. A previous study demonstrated that all examined tissues have a dramatic tissue specific transgenerational transcriptome change in the F3 generation [22]. In addition, several specific cell types examined (i.e. Sertoli cell and granulosa cell) have cell specific transgenerational transcriptome alterations in the F3 generation vinclozolin lineage animals [20, 21]. In considering the role of the DMR and sperm epimutations the observation was made that these transgenerational differentially expressed genes clustered in regions of 2-5 megabases with many having the DMR present, and these regions were termed epigenetic control regions [22]. Similar observations were made with somatic cell transgenerational transcriptome changes [20, 21]. In considering the epigenetic transgenerational inheritance of germline epimutations, the embryonic stem cells derived from these germ cells will have an abnormal epigenome. This suggests all cell types and tissues derived from the embryonic stem cells will have an altered epigenome and transcriptome [22]. Any tissue sensitive to this altered transgenerational transcriptome will have a susceptibility to develop disease [23].

Observations suggest these epimutations have a genomic feature of CpG deserts that are speculated to have significant roles in regulating genome activity [22]. Somatic cells have also been shown to contain epimutations and these DMR were generally distinct from the germline epimutations [20, 21]. Interestingly, these somatic epimutations also were present in CpG desert regions with small CpG clusters [20, 21]. Combined observations indicate the transgenerational epimutations primarily appear to be present in CpG deserts with small clusters of CpG in the DMR.

These DMR were previously shown to be exposure specific and had negligible overlap [13, 20, 21], Figure 1. The DMR ranged from 500 to 2000 bp with a density of <10 CpG/100 bp and no CpG islands were observed, only small clusters of CpG, Figures 3 and 4. Therefore, these CpG deserts do not appear to be CpG island shores, [6] but are distinct. The CpG genomic maps of specific CpG deserts that had the DMR confirmed with bisulfite mass spectrometry suggested the small clusters of CpG may be an important functional part of the CpG desert. The “CpG” desert is defined as a 500 bp to 2000 bp differential DNA methylation region with <15 CpG/100 bp and the presence of small CpG clusters, and the absence of CpG islands.

In considering the regulatory role and origins of these CpG deserts several previous observations were used. The first is that C to T conversions are the most frequent genetic point mutation, small nucleotide polymorphism (SNP), known to occur in a nearly order of magnitude greater frequency than any other SNP [24]. Therefore, evolutionarily this will lead to low density CpG regions developing in the genome. These low density CpG regions of the genome have been previously observed. In the event a cluster of CpG had a critical regulatory role for genome activity, these sites would be evolutionarily maintained as clusters of CpG within a CpG desert. Therefore, CpG deserts may have important roles in regulating gene expression [22]. This speculation of the regulatory role of these DMR now needs to be further investigated.

## Conclusions

In addition to the existing knowledge on the role of high density CpG islands and shores on the regulation of genome activity, the characterization of the transgenerational sperm and somatic epimutations suggests the importance of low density CpG regions, termed CpG deserts. Attention should be placed in the future on CpG deserts, in particular when studies address epigenetic transgenerational inheritance phenomenon. The advantage of next generation sequencing, bisulfite conversion of CpG sites, and new bioinformatics tools will likely advance this area quickly. Previous studies have also suggested low density CpG regions appear to be regulatory for genome activity [7, 9]. The speculation is made that these CpG deserts with small clusters of CpG in DMR will have a critical role in the epigenetic regulation of genome activity.

These CpG deserts are initially identified through characterization of the sperm epimutations that mediate the environmentally induced epigenetic transgenerational inheritance of disease and phenotypic variation [13, 14]. The transgenerational somatic epimutations also have a similar genomic feature [20, 21]. The similarities or differences in epimutations and genomic features such as the CpG desert between the F1, F2 and F3 generations remains to be investigated. However, the F3 generation data presented reflects the transgenerational germline epimutations [25]. Although these sites appear to be critical to the molecular mechanisms of epigenetic transgenerational inheritance, these CpG deserts are anticipated to have a much wider role in regulating genome activity associated with a large number of other biological phenomena and mechanisms.

## Methods

The environmentally induced epigenetic transgenerational inheritance of disease used an outbred Sprague Dawley rat model and the exposure of gestating females during fetal gonadal development as previously described [11–13]. The transgenerational F3 generation animals were used to isolate sperm or somatic cells as previously described [11, 13, 20, 21]. The procedure used to identify the DMR was a methylated DNA immunoprecipitation (MeDIP) followed by a genome wide promoter tiling array (MeDIP-Chip) as previously described [12, 13]. An individual DMR required a statistically significant difference between the F3 generation control lineage versus F3 generation exposure lineage sperm (p < 10^-5^) [13–19]. A minimum of three adjacent probes on the tiling array had to have the same statistical difference to identify the DMR. All the DMR used in the current report were previously published [12–21]. The specific DMR mapped in the current study were confirmed with a bisulfite mass spectrometry protocol previously described [12]. The statistical analysis for the identification involved R-code and significance (p < 10^-5^) being assigned to probe differences between treatment generation and exposure by calculating the median value of the intensity differences as compared to a normal distribution scaled to the experimental mean and standard deviation [13–19].

## Abbreviations

CpG: Cytosines adjacent to guanine
DMR: DNA methylation region
MeDIP: Methylated DNA immunoprecipitation
SNP: Small nucleotide polymorphism.
